# Doxorubicin-loaded platelets as a smart drug delivery system: An improved therapy for lymphoma

**DOI:** 10.1038/srep42632

**Published:** 2017-02-15

**Authors:** Peipei Xu, Huaqin Zuo, Bing Chen, Ruju Wang, Arsalan Ahmed, Yong Hu, Jian Ouyang

**Affiliations:** 1 Department of Hematology, Drum Tower Hospital, School of Medicine, Nanjing University, Nanjing, Jiangsu, 210093, P. R. China; 2 Interdisciplinary Research Centre in Biomedical Materials, COMSATS Institute of Information Technology, Lahore 54000, Pakistan; 3 Institute of Materials Engineering, Collaborative Innovation Center of Chemistry for Life Sciences, College of Engineering and Applied Sciences, Nanjing University, Nanjing, Jiangsu, 210093, China

## Abstract

Chemotherapy is majorly used for the treatment of many cancers, including lymphoma. However, cytotoxic drugs, utilized in chemotherapy, can induce various side effects on normal tissues because of their non-specific distribution in the body. Natural platelets are used as drug carriers because of their biocompatibility and specific targeting to vascular disorders, such as cancer, inflammation, and thrombosis. In this work, doxorubicin (DOX) was loaded in natural platelets for treatment of lymphoma. Results showed that DOX was loaded into platelets with high drug loading and encapsulation efficiency. DOX did not significantly induce morphological and functional changes in platelets. DOX-platelet facilitated intracellular drug accumulation through “tumor cell-induced platelet aggregation” and released DOX into the medium in a pH-controlled manner. This phenomenon reduced the adverse effects and enhanced the therapeutic efficacy. The growth inhibition of lymphoma Raji cells was enhanced, and the cardiotoxicity of DOX was reduced when DOX was loaded in platelets. DOX-platelet improved the anti-tumor activity of DOX by regulating the expression of apoptosis-related genes. Thus, platelets can serve as potential drug carriers to deliver DOX for clinical treatment of lymphoma.

Lymphoma, a hematologic malignancy, is a leading cause of cancer-related deaths and is mainly treated through chemotherapy^1^,^2^. However, minimizing the adverse effects of chemotherapy is still challenging. Doxorubicin is an effective chemo-therapeutic drug against lymphoma but elicits adverse side effects, including short biological life time, dose-dependent side effects, and cardiac toxicity induced by its nonspecific bio-distribution; these limitations restrict the successful clinical application of this drug^3^.

Scholars have developed several kinds of drug delivery systems, such as liposomes^4^,^5^, polymeric nanoparticles^6^,^7^, and polymeric micelles^8^,^9^ to achieve targeted therapy by delivering DOX to desired cells or tissues; these systems alleviate the side effects of DOX and enhance the efficacy of chemotherapeutics. Among these systems, liposomes were once considered the most successful drug carriers. However, limited drug loading, poor shelf stability, complicated synthesis, and high cost restrict the clinical application of liposomes^10^. Polymeric nanoparticles (NPs) or polymeric micelles can load different drugs flexibly and passively or actively, deliver them to the targeting tumor site to improve the therapeutic index against the tumor, and avoid multi-drug resistance in cancer cells^11^,^12^. These systems exhibit several shortcomings, such as nanotoxicity, limited biodegradability, adverse immune responses^13^, and short *in vivo* circulation time (several hours but longer than that of free drugs^14^). Moreover, the clinical applications of these systems are poorly understood^15^,^16^.

Cells or cell membrane derived from drug carriers, including erythrocytes, leukocytes, platelets, and stem cells, are extensively studied as a promising strategy to achieve ideal drug delivery systems that minimally interact with normal cells, target desired cells, and release drugs in a controlled manner; these systems exhibit good biocompatibility, good biodegradability, and immune evasion^17^. Platelets or platelet membranes enveloped in nanoparticles have gained increasing interest^18^ because of their innate function of targeting vascular disorders (such as cancer, inflammation, thrombosis, and hemorrhage^19^).

Platelets are small, enucleated, subcellular fragments derived from megakaryocytes of bone marrow^20^. The biocompatibility of platelets is superior over other drug carriers. Approximately 2 × 10^11^ to 5 × 10^11^ platelets are daily produced in an adult, and the number of platelets increase when the demand increases^21^. Thus, numerous platelets are produced. Platelets have an average lifetime of 7–10 days *in vivo*, which is suitable to be used as drug carriers, and are then removed by reticuloendothelial cells in the liver and spleen. Platelets with encapsulated agents demonstrate a systemic clearance similar to that of the natural platelets^21^. Thus, loaded drugs are protected and cloaked from immune surveillance and physical clearance, which provides prolonged circulation in blood. In addition, platelets play a vital role in hemostasis, inflammation, angiogenesis, wound healing, and thromboembolic diseases^22^,^23^. Previous study reported a close relationship between malignancy and platelets. The activation and adherence of platelets to the tumor cells are referred as tumor cell-induced platelet aggregation (TCIPA)^24^. Activated platelets release plenty of granular matter, and the loaded drugs are also released to the tumor site. Novel drug delivery systems are designed primarily based on the ability of platelets to adhere to tumor cells and deliver toxic drugs to targeted malignant cells. However, most of the existing platelet derived drug delivery systems are platelet-mimic by utilizing platelet membrane with complex process. The existence of foreign materials inside the drug carriers, such as platelet membrane-cloaked nanoparticles, is still a big challenge that brings adverse immune-response^25^.

In this study, DOX was loaded inside the platelets through the open canalicular system^26^ to treat lymphoma and achieve a longer retention time than synthetic drug delivery systems^27^. Given their excellent biocompatibility and immunocompatibility, platelets help DOX to escape from immunosurveillance and specifically deliver the DOX to tumor cells through TCIPA; this phenomenon greatly enhances the therapeutic efficacy, as well as reduces the drug dosage and damage to normal tissues^24^,^28^. The chemotherapeutic efficacy of DOX-loaded platelets (DOX-platelet) against lymphoma and their toxicity are evaluated in *in vitro* and *in vivo* experiments. Additionally, the possible mechanism of their anti-tumor activity is depicted in Fig. 1.

## Results

### Characterization of DOX-platelet

**Figure 2:**
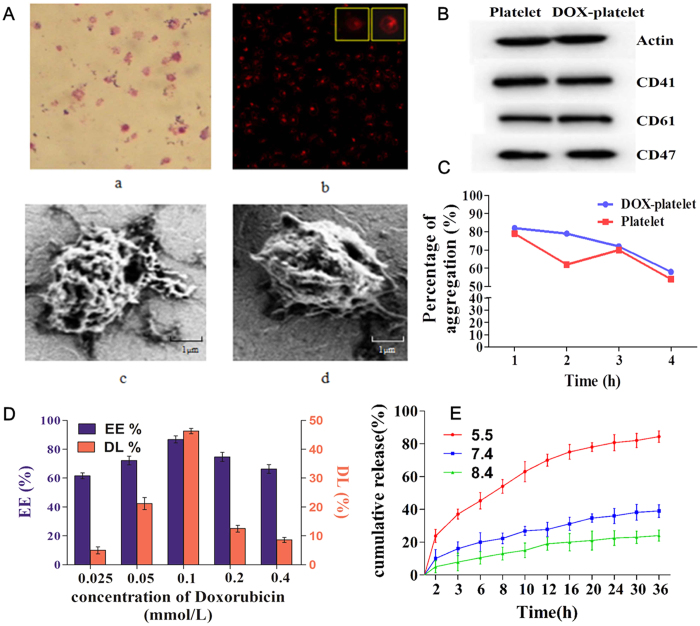
(**A**) a Image of platelets under oil immersion lens (400× ); b Image of platelets with 0.1 mmol/L DOX under fluorescence microscope (400×); c image of platelets under SEM; d image of platelets with o.1 mmol/L DOX under SEM. (**B**) Representative protein bands of platelets in western blotting. (**C**) Collagen-induced aggregation pattern of washed platelet and DOX-platelet at different time interval. (**D**) EE and DL of DOX-platelet with different incubation concentrations of DOX (0.025 mmol/L, 0.05 mmol/L, 0.1 mmol/L, 0.2 mmol/L, 0.4 mmol/L). (**E**) *In vitro* DOX release behaviors in PBS with different pH values (5.5, 7.4 and 8.4) at 37 °C respectively.

The encapsulation of DOX in platelets was first verified by the fluorescence microscopy as shown in Fig. 2A. Figure 2A(a) shows the typical morphology of platelets with irregular structure. After the encapsulation of DOX, red fluorescence related to the DOX was clearly observed inside these platelets, which confirm the encapsulation of DOX in the platelets (Fig. 2A(b)). The morphological changes in platelets before and after loading the DOX were observed by scanning electron microscopy (SEM) (Fig. 2A(c and d)). No significant change was observed between these two samples, which reveals that DOX has minimal influence on the morphology of platelets.

Translocation of platelet membrane protein content, including CD41, CD47, and CD61, was examined by Western blotting before and after DOX loading (Fig. 2B) to confirm that the platelets maintain their integrity and biological functions after the encapsulation. The content of platelet membrane proteins of DOX-platelet had no significant changes compared with that of natural platelets. This finding reveals that DOX-platelet retains the natural properties, which is the basis for platelets as drug delivery systems. The stability of DOX-platelet (blue line) and platelets (red line) in the buffer was tested, and their aggregation behaviors at different time points (1, 2, 3 and 4 h) are listed in Fig. 2C. The percentage of the aggregation of platelet at 1, 2, 3 and 4 h is 79%, 62%, 70%, and 54% for platelet and 82%, 79%, 72% and 58% for DOX-platelet, respectively. These results represent the loss of functionality of platelet and DOX-platelet is in a time-dependent manner. The average slopes of these two lines, i.e. the average loss of functionality line of platelet and DOX-platelet, are similar.

The DOX loading ability of platelets was measured. Different proportions of platelets to DOX were used to synthesize the optimized DOX-platelet drug delivery system (Fig. 2D). According to the fluorescence images, most DOXs were loaded in the platelets when the volume ratio of platelet to DOX was 1:2. Optimal drug loading (DL) (46.3%) and encapsulation efficiency (EE) (86.6%) were obtained at this volume ratio. Therefore, this dosage was selected in the following experiments. The *in vitro* release of DOX over time was analyzed and the results are listed in Fig. 2E. The cumulative release of DOX from DOX-platelet was pH sensitive. The most rapid release of DOX was obtained at pH 5.5, and approximately 84.4% of the loaded DOX was released into the buffer within 36 h. The release was slower at pH 7.4 and 8.4, suggesting a pH-triggered release pattern. The pH sensitive releasing ability of DOX is interesting because an acidic environment is found in tumor tissue (pH 6.8) or in tumor cells (pH 4–5), which describes that DOX is rapidly released from the DOX-platelet complex in the tumor or inside the tumor cells.

### Toxicity studies

**Figure 3:**
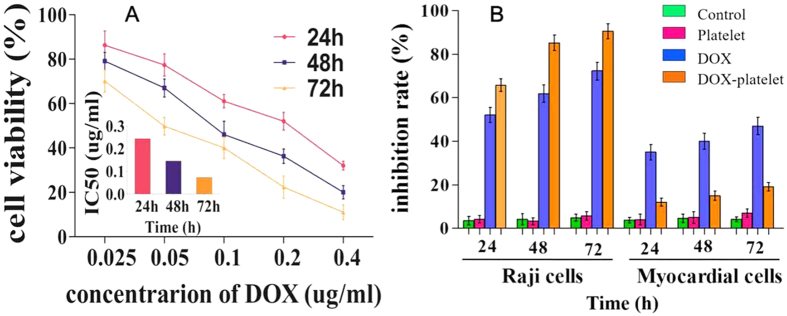
Cytotoxic effect of DOX-platelet. (**A**) The cytotoxic effect of DOX-platelet against Raji cells at 24, 48 and 72 h with different concentration of DOX; Inset: The IC_50_ of DOX inside the platelets for Raji cells at 24, 48 and 72 h. (**B**) Cytotoxic effect of platelet, free DOX and DOX-platelet on both Raji cells and myocardial cells at 24, 48 and 72 h. (P < 0.05 in the groups of DOX and DOX-platelet in comparison with controls at 24, 48 and 72 h both in Raji cells and myocardial cells).

The viability of Raji cells, a lymphoblastoid cell derived from a Burkitt lymphoma, treated with DOX-platelet containing different concentrations of DOX (0.025, 0.05, 0.1, 0.2, and 0.4 μg/ml) was studied to estimate the anti-tumor effect of DOX-platelet on lymphoma (Fig. 3A). The IC50 of DOX at 24 h was 0.243 μg/ml. Considering the DL of DOX-platelet, 0.524 μg/ml of DOX-platelet (equivalent to 0.243 μg/ml of DOX) was selected to measure their cytotoxicity against the Raji cells with different incubation times. As illustrated in Fig. 3B, the cytotoxicity of DOX-platelet against Raji cells is positively related to the incubation time, which is also higher than that of free DOX within 72 h. After incubation for 24, 48, and 72 h, the corresponding inhibition rates of DOX were 52.06%, 61.90%, and 72.31%, whereas that of DOX-platelet were 65.8%, 85.23%, and 90.6%, respectively. A significant difference was observed between the two groups at 24, 48, and 72 h (P < 0.05). However, the blank platelets showed no significant reduction in cellular viability compared with control, revealing the good biocompatibility of platelets to the cells.

DOX causes severe cardiotoxicity that greatly restricts its application in tumor therapy. In this study, the toxicities of free DOX and DOX-platelet against myocardial cells were tested by the CCK-8 assay. The viability of myocardial cells decreased when incubated with DOX for a longer time, confirming the time-dependent *in vitro* cardiotoxicity of DOX. More than 80% of the cells treated with DOX-platelet survived even after 72 h. These results reveal that DOX-platelet exerts high antitumor effect and low cardiotoxicity.

### Raji cell uptake and apoptosis

**Figure 4:**
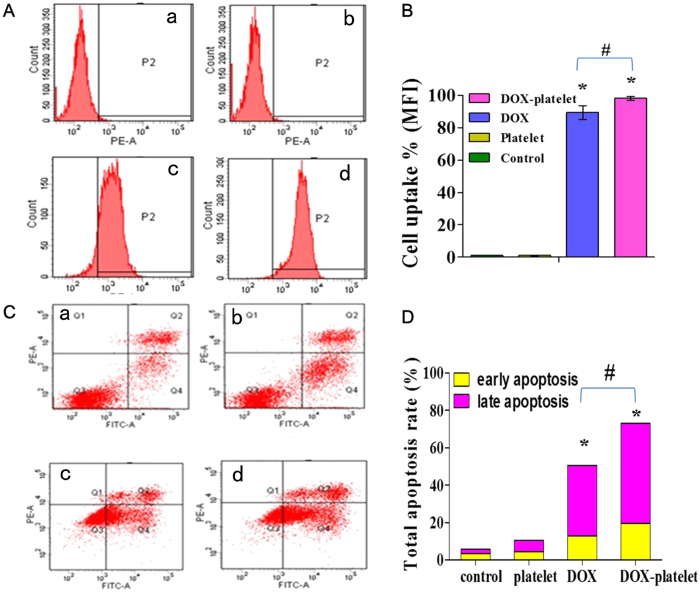
Mean fluorescence intensity (MFI) in Raji cells and apoptosis of Raji cells. (**A**) FCM was utilized to figure out the intracellular uptake of DOX in Raji cells with different treatments (a = control; b = platelet; c = DOX; d = DOX-platelet). (**B**) Intracellular uptake of DOX in Raji cells of different sampless. (**C**) The apoptosis of Raji cells (a = control; b = treated with platelet; c = treated with DOX; d = treated with DOX-platelet). (**D**) Quantitative data of apoptosis from (**C**) (*P < 0.05 when compared with controls, ^#^P < 0.05).

The accumulation of DOX in Raji cells was measured by flow cytometry (FCM) to determine whether the cytotoxic activity of DOX or DOX-platelet is related to the intracellular DOX level in Raji cells. As shown in Fig. 4A and B, the intracellular DOX concentration significantly increased in the DOX-platelet group compared with that in the free DOX group, which positively correlates with the cytotoxic activity. FCM was also used to quantitatively investigate the apoptosis of Raji cells (Fig. 4C and D). After the incubation for 24 h, the total apoptosis rate of the controls was 5.9%, including the early and late apoptosis rates of 3.4% and 2.5%, respectively. Similarly, the early and late apoptosis rates of platelet-treated cells were 8.6% and 6.0%, respectively, indicating no significant difference with the controls. However, the total apoptosis rate of the DOX treated groups increased to 50.5% after 24 h. When the cells were treated with DOX-platelet, the early and late apoptosis rates increased to 19.5% and 53.6%, respectively, which confirms an enhanced cellular apoptosis compared with that in the DOX treated cells (P < 0.05).

**Figure 5:**
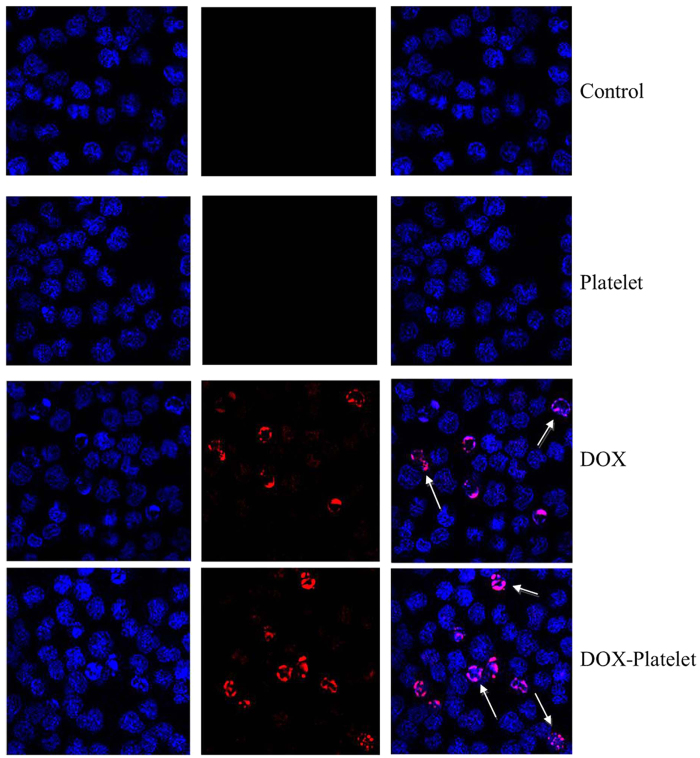
The fluorescence microscopy images of Raji cells after receiving different treatment (400×). The nucleus was stained by DAPI. Cell apoptosis is indicated by arrows.

Fluorescence microscopy was then used to observe the morphological changes in Raji cells stained with DAPI. As shown in Fig. 5, the control cells were homogenously stained with blue fluorescence, revealing that the chromatin was equally distributed in the nucleolus. The cells treated with platelets showed no significant morphological changes compared with the controls. When these cells were treated with DOX, the signal of cell apoptosis was observed, such as chromatin condensation, nucleolus pyknosis, and nuclear fragmentation. The morphological changes were also more drastic when the DOX-platelet was used against the Raji cells. These results confirm that DOX induces the apoptosis of Raji cell. In addition, the uptake of DOX by the Raji cells is strengthened by DOX-platelet, which consequently improves the cytotoxicity against the cells.

### RT-PCR and Western blot assay of Raji cells

**Figure 6:**
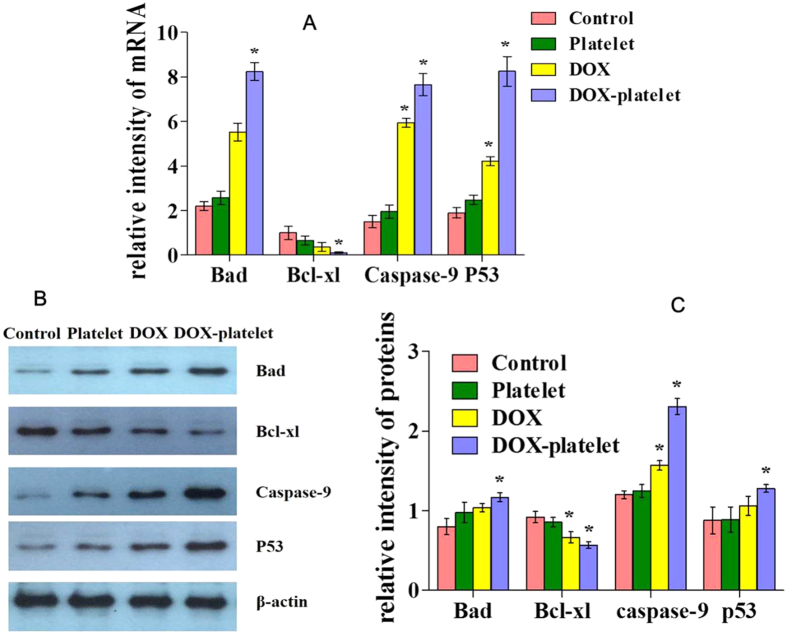
(**A**) mRNA expression of apoptosis-associated genes with different treatments evaluated by RT-PCR. (**B**) and (**C**): Protein expression of apoptosis-associated genes by western blotting (*P < 0.05).

The expression levels of Bad, Bcl-xl, caspase-9, and p53 were detected using RT-PCR. Western blot was conducted to evaluate the mechanisms leading to the apoptosis of Raji cell. As shown in Fig. 6A, the mRNA expression of Bad, caspase-9, and p53 was slightly upregulated in the DOX treated group compared with that in the control group. The level of these mRNAs was greatly evaluated for the DOX-platelet treated cells (P < 0.05). However, the Bcl-xl transcription level was downregulated in the DOX group compared with that in the control, and the DOX-platelet treated group had the lowest level of Bcl-xl among the groups. The expression changes of all these apoptosis-related genes clearly indicate that DOX-platelet improves the anti-tumor activity of DOX against Raji cells by strengthening the cellular apoptosis. Figure 6B and C show the results of Western blot analysis. No significant up-regulation of caspase-9 protein was observed in the control and platelet treated groups, whereas the DOX-platelet group exhibited the highest level of caspase-9, which also confirms the serious apoptosis of the Raji cells.

### Macrophage uptake in livers and spleens *in vivo*

**Figure 7:**
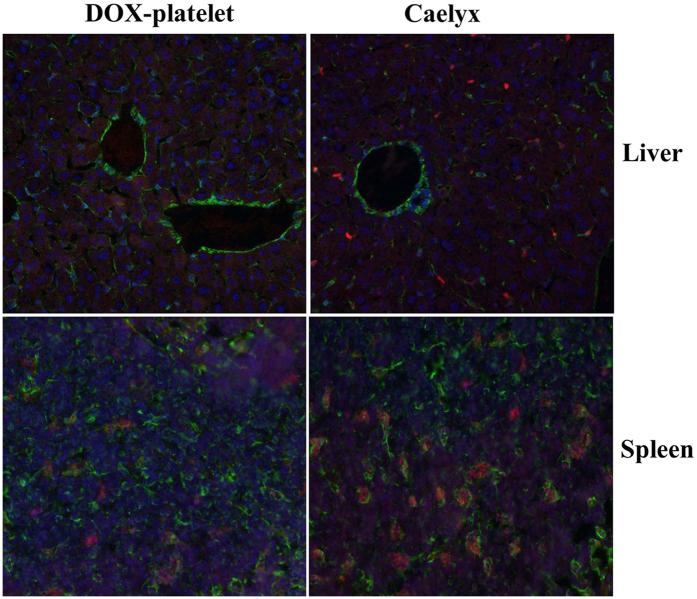
Confocal fluorescence images of liver and spleen tissues after injection with DOX-platelet or Caelyx (200×). The nuclei were stained by DAPI. Cytoskeletons were labeled with FITC.

As shown in Fig. 7, the macrophages in livers and spleens from the Caelyx-treated mice have significantly enhanced red fluorescence density of DOX compared with those of the DOX-platelet-treated mice. In contrast, lower fluorescence density of DOX was observed in the livers and spleens of DOX-platelet-treated mice. The results reveal that less DOX in the form of DOX-platelet was captured by macrophages than in the form of pegylated liposomal DOX(Caelyx), which suggests that the immune compatibility of DOX-platelet is superior to that of pegylated liposomal DOX.

### Plasma concentration and tissue distribution of DOX *in vivo*

**Figure 8:**
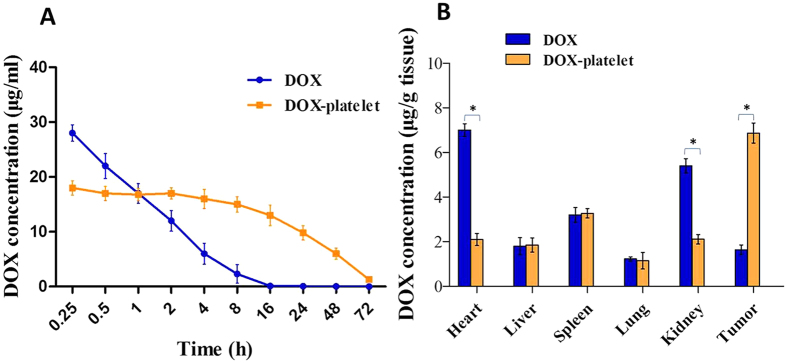
Plasma concentration and tissue distribution of DOX *in vivo*. (**A**) Plasma concentrations of DOX over time after injection with free DOX or DOX-platelet. (**B**) Tissue distribution of DOX at 4 h after injection with free DOX or DOX-platelet (*P < 0.05).

The DOX concentration in plasma over time after injection is shown in Fig. 8. Based on the figure, free DOX was rapidly cleared out from the blood with a short half-life (t_1/2_ = 1.89 ± 0.53 h), whereas DOX-platelet had a prolonged blood circulation time with a long half-time (t_1/2_ = 29.12 ± 1.13 h) (Fig. 8A). Results of tissue distribution is are shown in Fig. 8B. The DOX concentration from mice injected with free DOX was high in the heart tissue but low in the tumor tissue. On the contrary, the heart tissue in the DOX-platelet-treated mice had a significantly lower concentration of DOX than that in the mice treated with free DOX (P < 0.05). Moreover, the tumor tissue of mice treated with DOX-platelet had higher concentration of DOX than that with free DOX (P < 0.05). Additionally, the liver, spleen, kidney and lung tissues had low DOX concentration. These results reveal that DOX-platelet, with a slow rate of clearance from circulation, can effectively accumulates and releases DOX in the tumor tissues and protect the normal tissues from toxicity caused by high DOX concentration.

### Therapeutic efficacy *in vivo*

**Figure 9:**
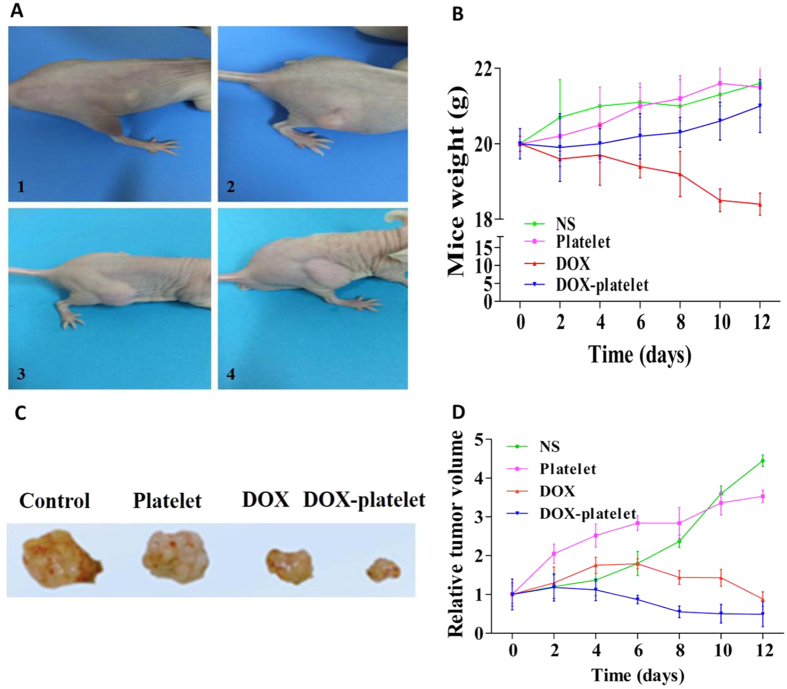
(**A**) The establishment of tumor-bearing mouse models (from 1 to 4). (**B**) The body weight changes of mice in the period of 12 days after different treatments. (**C**) The final tumor size of mice with different treatments. (**D**) The changes of relative tumor volume of mice in the period of 12 days after different treatments.

The tumor-bearing mouse model was established to investigate the *in vivo* anti-tumor effects of DOX-platelet (Fig. 9A). During the treatment, the body weight and tumor size of mice were monitored every 2 days. As shown in Fig. 9B, compared with the initial body weight (20 g), the final body weight of mice in the groups treated with normal saline, platelet, and DOX-platelet was 21.6, 21.5, and 21 g, respectively. However, after the treatment with free DOX for 12 days, the mice have a final average weight of 18.4 g, suggesting a remarkable weight loss compared with the controls. This finding reveals that the toxicity of free DOX against normal tissue is serious and is greatly reduced by the encapsulation of platelet. The tumor size of mice treated with saline or platelet alone continuously increased, whereas for the groups treated with free DOX, the tumor size increased during the first 4 days, followed by a size decrease (Fig. 9D). For the DOX-platelet treated group, a continuous tumor suppression was observed during the whole experiment (Fig. 7D), and the smallest tumor size was achieved at the end of the experiment (Fig. 9C).

### Histopathological study

**Figure 10:**
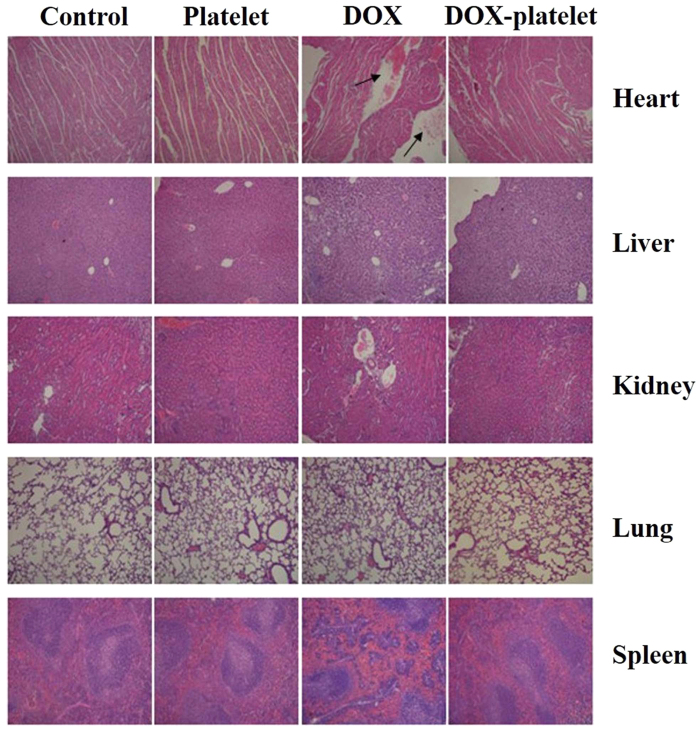
The H&E staining images of the sections of heart, liver, kidney, lung and spleen (100×). The histopathological changes are indicated by arrows.

The histopathological changes on major organs (heart, liver, spleen, lung, and kidney) of mice after platelet, DOX, and DOX-platelet treatments were conducted by hematoxylin-eosin (H&E) staining and are shown in Fig. 10. After saline, platelet, and DOX-platelet treatments, no significant histopathological abnormalities, such as morphological or structural changes, hemorrhage, necrosis, and inflammatory exudates, were observed on any organ. However, typical DOX-induced myocardial injury to the mouse heart, which is related to acute inflammatory cells, was observed in the DOX treated sample. These results suggest that the platelet protects the heart from the direct exposure to DOX and therefore reduce its side effect.

### RT-PCR and Western blot assay of tumor bodies

**Figure 11:**
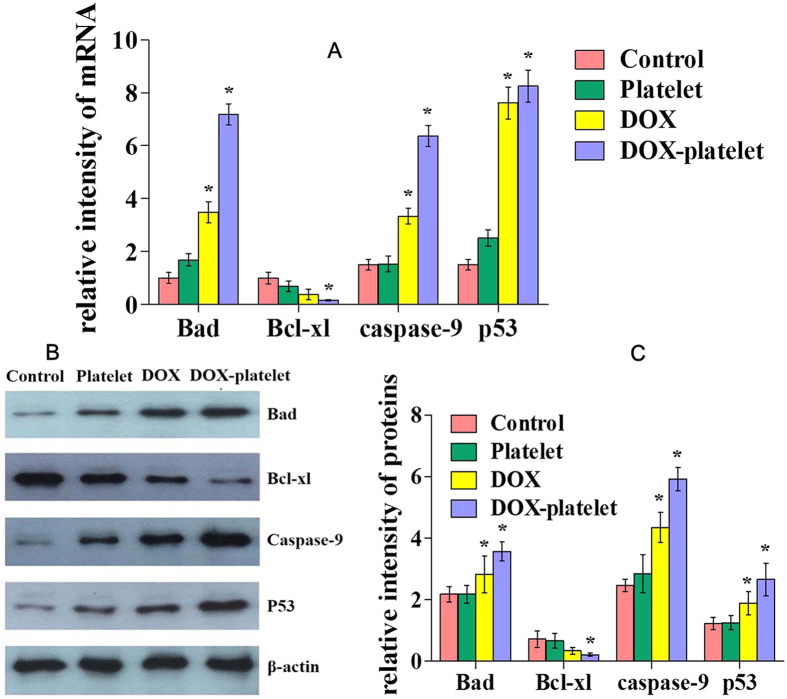
(**A**) mRNA expression of apoptosis-associated genes with different treatments evaluated by RT-PCR. (**B**) and (**C**): Protein expression of apoptosis associated genes by western blotting (*P < 0.05).

RT-PCR and Western blot were performed to evaluate the expression levels of Bad, Bcl-xl, caspase-9 and p53 in tumor cells *in vivo*. As shown in Fig. 11, the highest mRNA expressions of Bad, caspase-9 and p53, as well as the lowest mRNA level of Bcl-xl were obtained in the group of DOX-platelet (Fig. 11A). The same variation tendency was also observed in the Western blot assay (Fig. 11B,C). These results are in accordance with the *in vitro* experiments, revealing that the DOX-platelet shows the best tumor inhibition.

## Discussion

Lymphoma is one of the most fatal diseases worldwide, and DOX is the first choice in the treatment of lymphoma. However, cytotoxic drugs such as DOX induce various adverse effects on normal tissues and organs. Therefore, the design of drug delivery systems to alleviate the side effects and improve the therapeutic index has been desired during the past several decades. Biocompatibility is important for a practical drug delivery system to prevent unwanted immune response and tissue reactions against the drug carrier^29^. Although many materials were used for drug delivery systems, the problem of biocompatibility hindered their broad clinical application. Platelets attracted attention as a potential biological drug delivery system, but the existing studies either mimic the function of platelets or use the platelet membrane to cloak the particles^25^. All these studies are subjected to complicated procedures, and natural platelets are currently seldom used as drug carriers. In this study, we developed a DOX-loaded delivery system using platelets as the starting material because of their unique biocompatibility.

Platelets encapsulate drugs with relatively high DL and EE as reported in several studies. Sarkar *et al*. reported that approximately 10^13^ molecules of drugs were contained per platelet^30^. Our study revealed that platelets load DOX with high DL and EE without including morphological and functional changes. The study of macrophage uptake *in vivo* directly revealed that DOX-platelet has a more favorable immunocompatibility than pegylated liposomal DOX and reduces the uptake by macrophages (Fig. 7). Thus, the blood circulation time increased with a high half-life. As illustrated in Fig. 2E, more DOX was released at low pH, indicating a pH-triggered release pattern. Tumor microenvironment has a lower pH compared with normal tissues because of the hypoxic states^31^. Thus, the substantial release of drugs occurs around the tumor cells, whereas most drugs remain in the carriers in the normal physiological environment, leading to less exposure of normal tissues from cytotoxic drugs. The intracellular studies also confirmed that more drugs were delivered to Raji cells. Therefore, the chemotherapeutic efficacy was enhanced and the undesirable adverse effects on normal tissues were attenuated.

The growth inhibition and apoptosis of Raji cells were enhanced with DOX encapsulated in platelets compared with those with the free DOX at the same concentration. Therefore, less DOX is required to achieve the same chemotherapeutic efficiency. This finding suggests that normal tissues are exposed to less amount of DOX, and the side effects induced by DOX are alleviated. The typical adverse effects of DOX include myelosuppression, nausea, vomiting, and cardiotoxicity. Among these effects, cardiotoxicity seriously restricts the practical application of DOX. The present study evaluated the toxicity of DOX and DOX-platelet against myocardial cells. As illustrated in Fig. 3B, minimal toxic effect on myocardial cells was detected when using the DOX-platelet *in vitro*, confirming that the platelets alleviate the cardiotoxicity of DOX. Cardiotoxicity caused by DOX is dose-dependent. It occurs when the dose of DOX exceeds a certain threshold level. Since DOX-platelet is not activated when cultured with myocardial cells and only 30% of DOX is released and diluted in culture media, the dose of DOX might not reach the threshold level that can cause cardiotoxicity. These results are consistent with the tissue distribution of DOX-platelet *in vivo*, which reveals that more DOX accumulate in tumor tissues whereas less DOX accumulate in normal tissues (Fig. 8).

*In vivo* therapeutic efficacy and toxicity were further estimated using a tumor-bearing mouse model. Figure 10 shows that the tumor size in both DOX and DOX-platelet treated groups was significantly decreased compared with that in controls, which illustrates the super efficiency of DOX for lymphoma therapy. Moreover, the mice treated with DOX-platelet exhibited the smallest tumor size among the four groups, suggesting that DOX-platelet is more effective than free DOX in the cancer therapy. Therefore, DOX-platelets specifically target the tumor tissues while producing less side effects caused by DOX. The most serious adverse effect of DOX is myocardial injury. In the H&E staining experiments, significant myocardial injury was only observed in the free DOX treated group, revealing that DOX-platelet reduces the cardiotoxicity of DOX. As a clinical symptom, body weight loss is an early indicator for side effects. In our study, body weight loss was discovered only in the group receiving the treatment of free DOX. These results confirm that when platelets act as drug carriers, the therapeutic efficacy of DOX is improved and the side effects are reduced.

Apoptosis in mammalian cells consists of two main pathways, the death receptor-mediated (extrinsic) and the mitochondrial-mediated (intrinsic) pathways. The extrinsic pathway is mediated by the binding of ligands to corresponding death receptors^32^. The intrinsic pathway is the release of apoptogenic factors, such as cytochrome C, from the mitochondria into the cytosol. Once cytochrome C forms an apoptosome with apoptosis-activating factor 1 and caspase-9, the downstream apoptotic signals are activated^33^. Growing evidences suggest that the intrinsic apoptosis pathway is regulated by the Bcl-2 family^34^, which is the apex of the “life or death” cellular mechanisms. The Bcl-2 family consists of pro-apoptotic (Bax, Bad, and Bak) and anti-apoptotic (Bcl-xl and Bcl-2) proteins^35^. The expression of Bcl-xl or Bcl-2 is relatively high in more than 50% of all cancers^36^. Ranger *et al*. found that Bad-deficient mice progressed to diffuse large B cell lymphoma of germinal center origin^37^. In our study, Bcl-xl was clearly downregulated when the DOX-platelet was used. By contrast, the expression level of Bad increased in both the free DOX and DOX-platelet treated groups. Caspase-9 also plays a vital role in the intrinsic apoptosis pathway. When caspase-9 is activated, the effector caspases are cleaved and activated, leading to lysis of numerous cellular substrates and cell death^38^. Our data revealed the increased expression of mRNA and protein of caspase-9 after the DOX treatment both *in vitro* and *in vivo*. This increment is more pronounced in the DOX-platelet treated group. These results reveal that the pro-apoptotic pathway promotes the apoptosis by inhibiting the anti-apoptotic Bcl-2 proteins through heterodimerization, thereby leading to the release of cytochrome C and the activation of the caspase pathway^36^. P53 is another tumor-suppressor gene and its expression is increased in both free DOX and DOX-platelet treated groups. P53 regulates the expression of some downstream apoptosis genes, such as Bax and Bcl-2^39^. The loss of p53 commonly occurs in malignancy and triggers an escape route from apoptosis^40^. However, the relationship between p53 and Bad or Bcl-xl is poorly understood. In our study, the expressions of Bad, caspase-9, and p53 were upregulated and the expression of Bcl-xl was downregulated in both DOX and DOX-platelet treated groups, especially in the latter. According to these results, DOX induces tumor cell apoptosis by regulating the expression of apoptosis-related genes. Furthermore, evident changes of their expression were observed in DOX-platelet treated group, which reveals that DOX-platelet improves the anti-tumor activity of DOX by enhancing DOX-induced cell apoptosis.

## Materials and Methods

### Materials

#### Reagents

DOX was purchased from Dalian Meilun Biology Technology Co., China. Roswell Park Memorial Institute (RPMI) 1640 medium was obtained from Thermo Fisher Scientific, Waltham, MA, USA. Cell counting kit-8 (CCK-8) assay, Annexin V-Fluorescein isothiocyanate (FITC) apoptosis detection kit, and 4′,6-diamidino-2-phenylindole (DAPI) were obtained from Beyotime Biotechnology Co., Ltd. (Nantong, China). Monoclonal antibodies to caspase-9, Bad, Bcl-xl, p53 and GAPDH were from Santa Cruz Biotechnology Inc (Santa Cruz, CA, USA). Hematoxylin-eosin staining (H and E staining) were bought from Thermo Fisher Scientific, Waltham, MA, USA. All other reagents were of analytical grade and without further purification.

#### Animals

All experimental protocols using mice were approved by the Medical Animal Care and Welfare Committee of the Affiliated Drum Tower Hospital of Nanjing University Medical School (Nanjing, China). The methods were carried out in accordance with the relevant guidelines, including any relevant details. The athymic BALB/c-nude mice (4–6 weeks, 18–22 g in body weight) were purchased from Shanghai Experimental Animal Center of Chinese Academic of Science and kept in specific pathogen free (SPF) conditions with controlled temperature (23 ± 2 °C) and humidity (60% ± 5%), a 12-h light/dark cycle, and free access to water and food.

### Preparation and characterization of DOX-platelet

#### Platelet isolation

Fresh human blood was obtained from healthy volunteers with permission from the Ethics Committee of the Affiliated Drum Tower Hospital of Nanjing University Medical School in accordance with the Declaration of Helsinki. All volunteers signed the informed consent before donating their blood for the study, and all experiments were performed in accordance with relevant guidelines and regulations. Briefly, 9 ml of blood and 1 ml of 3.2% sodium citrate anticoagulant were mixed and centrifuged at 200 × g for 10 min at room temperature to obtain platelet-rich plasma (PRP). Then PRP was centrifuged at 1800 × g for 20 min twice and the supernatant was discarded. The resulting pellet was washed with phosphate-buffered saline (PBS) to prepare purified platelets^41^. All experiments were performed under sterile conditions. Oil immersion microscopy was used to observe the morphology and distribution of platelets.

#### Drug loading

DOX was dissolved in PBS with different concentrations and mixed with platelets. The mixture was gently shaken and incubated at 37 °C for 1 h at 100 rpm in the dark. Thereafter, the mixture was passed through a Sepharose 2B column (Invitrogen, Carlsbad, CA, USA) to remove the free DOX. All steps were conducted under sterile conditions in the dark. Precautions were taken to prevent the activation of platelets.

#### Characterization of DOX-platelet

Fluorescence microscopy was performed to determine the encapsulated DOX in platelets. One drop of the DOX-platelet suspension was placed on a clean glass slide. The glass slide was observed under a fluorescence microscope. Morphological changes of DOX-platelet were investigated by SEM (JEM-2100, JEOL Ltd., Tokyo, Japan). Briefly, 200 μL of platelets before or after the encapsulation of DOX were dropped onto the glass. Gradient dehydration with 4% methyl alcohol was performed twice, and the samples were washed with PBS before observation.

Western blotting and platelet aggregation assay were performed to determine the platelet membrane proteins and evaluate the activation of DOX-platelet, respectively. Platelet aggregation was estimated by a spectrophotometric method. Briefly, 1 ml of platelet rich plasma that was anticoagulated with sodium citrate was mixed with 500 ml of PBS or 500 ml of PBS containing 0.5 IU/ml of thrombin, as negative and positive controls, respectively. The optical density at 650 nm of each group was monitored over time to measure the platelet aggregation based on the reduction of turbidity.

DL and EE were measured by high-performance liquid chromatography (HPLC). Freshly prepared DOX-platelet suspension was centrifuged at 2000 rpm for 15 min, and the supernatant containing free DOX was collected and diluted with PBS for HPLC evaluation^42^.

Dynamic dialysis method was used to investigate the release behavior of DOX from the DOX-platelet *in vitro*. DOX-platelet was dispersed in 5 ml of PBS at pH 7.4 and was transferred to a dialysis bag immersed in 95 ml of PBS at different pH values of 5.5, 7.4, and 8.4. After shaking in a horizontal shaker at 100 rpm at 37 °C for predetermined time intervals, 2 ml of the external medium was collected and replaced with an equal volume of fresh PBS. The DOX concentration was determined by spectrophotometry at 450 nm, and the cumulative release of DOX from the platelets was plotted according to the release ratio over time.

### Anti-tumor activity and cardiotoxicity of DOX-platelet *in vitro*

#### Cell culture

Raji cells, a cell line of lymphoma cells, were cultured in a RPMI-1640 supplemented with 10% fetal bovine serum at 37 °C in a humidified atmosphere of 5% CO2. The cells were passed every 2–3 days to maintain the best state. Cells were treated with platelets, DOX, and DOX-platelet, and cells without treatment were labeled as controls. The myocardial cells were isolated from mouse hearts and used immediately after centrifugation and washing.

#### CCK-8 assay

The CCK-8 assay was used to evaluate cell viability. The cells were seeded in a 96-well plate with the concentration of 8 × 10^3^ cells per well. After incubation for 24, 48, and 72 h at 37 °C in a humidified atmosphere of 5% CO2, 10 μL of CCK-8 solution was added into each well and incubated for another 4 h. The absorbance values of the solution per well were determined using a spectrophotometer at 450 nm, and the cytotoxic activity was measured by reading the optical density (OD). Cell viability (%) was calculated as follows:


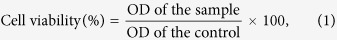


where OD of sample represents the optical density of cells treated with platelets, DOX and DOX-platelet, and OD of control describes the optical density of cells without treatment.

#### Cellular uptake

Cellular uptake was quantitatively measured using flow cytometry (FCM). Briefly, Raji cells were cultured in six-well plates and incubated for 24 h. After incubation for another 24 h with platelets, DOX, and DOX-platelet, these cells were centrifuged and collected at 1000 rpm for 5 min. The culture medium was discarded, and the precipitate was dispersed in 200 μL of PBS and incubated for 15 min in the dark. Finally, the cellular uptake was determined by FCM.

#### Raji cell apoptosis

Raji cells at a density of 6 × 10^5^ cells/well were cultured in a six-well plate overnight and treated with platelets, DOX, and DOX-platelet for 24 h. Afterwards, the cells were washed with cold PBS, followed by staining with 5 μL of Annexin V-FITC in binding buffer for 15 min in the dark. FCM was used to quantitatively detect cell apoptosis.

#### Morphological changes in Raji cells

Raji cells were cultured and collected as previously described. The cells were stained with DAPI and the morphological changes were observed with a fluorescence microscope.

#### Reverse transcription-polymerase chain reaction (RT-PCR) and Western blot analysis

The RT-PCR method was employed to determine the transcription levels of genes. Total RNA was extracted with TRIZOL reagent and transcribed using a Reverse Transcription System. To obtain cDNA, the reverse transcriptase solution was incubated at 42 °C for one hour, 85 °C for 5 minutes, and 5 °C for 5 minutes. The designed PCR primers included Bad primer (sense:5′-GTGACCTTCGCTCCAC ATC-3′, antisense: 5′-GAGACAGCACGGATCCTCTT-3′), Bcl-xl primer (sense: 5′-CTATGGGAACAATGCAGCAG-3′, antisense: 5′-TGGTCATTTCCGACTGAAG A-3′), caspase-9 primer (sense: 5′-AGACCCAGGTCCAGATGAAG-3′, antisense: 5′-TTTCTGGGAAGGGACAGAAG-3′), p53 primer (sense: 5′-TACATCTGGC CTTGAAACCA-3′, antisense: 5′-CAGCTGCCCAACTGTAGAAA-3′) and GAPDH primer (sense 5′-TGTTGCCATCAATGACCCCTT-3′, antisense 5′-CTCCACGACG TACTCAGCG-3′). The newly synthesized cDNA was amplified by PCR. Denaturation was performed at 95 °C for 2 minutes, and final extension at 72 °C for 10 minutes. RT-PCR products were analyzed using the ScnImage software (Scion Corporation, Frederick, MD).

After treatments, total proteins were isolated on ice, subjected to sodium dodecyl sulfate polyacrylamide gel electrophoresis, and transferred to a polyvinylidene difluoride membrane. After blocking with 5% nonfat milk for one hour at room temperature, the blots were stained with mouse monoclonal anti-human Bad, Bcl-xl, caspase-9, p53 or β-actin overnight at 4 °C and subsequently incubated with horseradish peroxidase-labeled immunoglobulin G as the secondary antibody. The blots were visualized using the enhanced chemiluminescence detection system (Amersham, UK), and β-actin was used as the internal control.

### Therapeutic efficacy of DOX-platelet *in vivo*

#### Establishment of tumor-bearing mouse models

Subcutaneous tumors were established by injecting 2 × 10^6^ cells/ml Raji cells in 200 μL of complete medium into the axillary subcutaneous space of each mouse. The tumor model was successfully established when the volume of tumor reached 60–100 mm^3^.

#### Macrophage uptake in livers and spleens in vivo

Caelyx (pegylated liposomal doxorubicin HCl; Schering-Plough) was used as the standard stealth liposome formulation of DOX. Tumor-bearing mice were intravenously administered with DOX-platelet or Caelyx (DOX 5 mg/kg). At 4 h post-injection, the mice were sacrificed and their livers and spleens were separated and prepared as frozen sections. For cytoskeleton staining, the sections were incubated with anti-vimentin antibodies overnight at 4 °C. IgG-FITC was added and incubated at 37 °C for another 1 h in the dark. Subsequently, cell nuclei were stained by DAPI. The sections were observed with a laser scanning confocal microscope.

#### Plasma concentration and tissue distribution of DOX in tumor-bearing mice

Tumor-bearing mice were intravenously injected with free DOX or DOX-platelets (DOX 5 mg/kg). Blood samples for measuring the DOX concentration were collected via heart puncture after 0.25, 0.5, 1, 2, 4, 8, 16, 24, 48, and 72 h. Heparin sodium was used as an anticoagulant. Plasma was obtained after the whole blood was centrifuged at 3000 rpm for 10 min. The DOX concentration in plasma was determined by spectrofluorometry with excitation and emission wavelengths of 485 and 590 nm, respectively.

The tumor bearing mice were sacrificed at 4 h after injection with a single dose of free DOX or DOX-platelets (DOX 5 mg/kg). Their main organs (liver, kidney, spleen, lung, and heart) and tumor tissues was isolated and washed with PBS. The tissues were lysed and homogenized. Acidified isopropanol was used to extract DOX from the tissue homogenate. After centrifugation, the supernatant was analyzed in a spectrofluorometer.

#### Study of the therapeutic efficacy

The tumor-bearing mice were divided into 4 groups with 6 mice in each group according to the solutions they were administered: (1) normal saline group; (2) platelet group; (3) DOX group; (4) DOX-platelet group. The dosage of DOX for each injection was 1 mg/kg, and according to the effective drug loading rate of 46.3%, the dose of DOX-platelet was 2.15 mg/kg (Fig. 2D). They were intravenously administered via the tail vein and the volumes of tumors were measured every 2 days. After 12 days, all the mice were sacrificed and the tumor, heart, liver, spleen, lung, and kidneys were isolated for further examinations. The volume (V) of tumor and relative tumor volume (RTV) were calculated as the following equations:


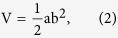


where a and b represent the longest and shortest vertical dimensions of the tumor respectively.


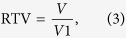


where V1 presents the tumor volume on the first day of treatment.

#### Reverse transcription-Polymerase chain reaction (RT-PCR) and Western blot assay

To measure the expression levels of mRNAs and proteins of Bad, Bcl-xl, caspase-9 and p53 in the tumor cells, RT-PCR and western blotting were carried out.

#### Histopathological analysis

The major organs of mice were isolated carefully, washed by PBS and then immersed into 4% paraformaldehyde solution at 4 °C overnight. Afterwards, they were embedded in paraffin blocks, sectioned with 5 μm thickness and placed onto glass slides. The slides were finally stained with H&E staining for histopathological examination.

### Statistical analysis

The results were expressed as the means ± standard deviation. Statistical analyses were performed with a parametric test (Student’s t-test) using the SPSS software. A P-value, less than 0.05, was considered statistically significant.

## Additional Information

**How to cite this article:** Xu, P. *et al*. Doxorubicin-loaded platelets as a smart drug delivery system: An improved therapy for lymphoma. *Sci. Rep.* **7**, 42632; doi: 10.1038/srep42632 (2017).

**Publisher's note:** Springer Nature remains neutral with regard to jurisdictional claims in published maps and institutional affiliations.

## Figures and Tables

**Figure 1:**
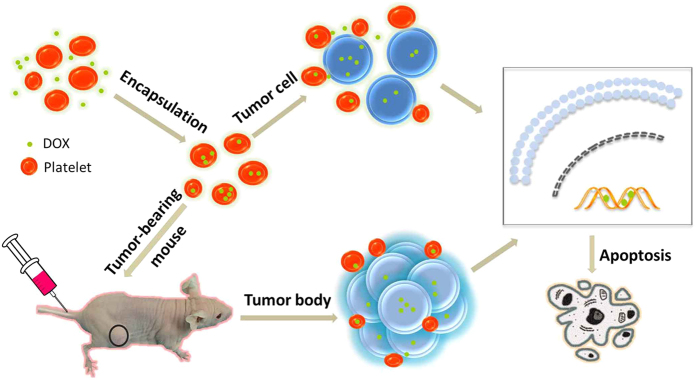
Schematic illustration of the possible mechanism of enhanced anti-tumor activity of DOX-platelet *in vitro* and *in vivo*. Tumor-bearing mice are injected with DOX-platelet by tail vein. DOX-platelet targets tumor cells passively by “tumor cell-induced platelet aggregation”. DOX induces tumor cell apoptosis through regulating the expression of apoptosis-related genes.
