# Corrigendum: Doxorubicin-loaded platelets as a smart drug delivery system: An improved therapy for lymphoma

**DOI:** 10.1038/srep44974

**Published:** 2017-03-28

**Authors:** Peipei Xu, Huaqin Zuo, Bing Chen, Ruju Wang, Arsalan Ahmed, Yong Hu, Jian Ouyang

Scientific Reports 7: Article number: 4263210.1038/srep42632; published online: 02 15 2017; updated: 03 28 2017

This Article contains a typographical error in the Results section, under the subheading “Therapeutic efficacy *in vivo*”, where:

“For the DOX-platelet treated group, a continuous tumor suppression was observed during the whole experiment (Fig. 7D)”.

should read:

“For the DOX-platelet treated group, a continuous tumor suppression was observed during the whole experiment (Fig. 9D)”.

